# Ozone and cardiovascular injury

**DOI:** 10.1186/1476-7120-7-30

**Published:** 2009-06-24

**Authors:** Vera Srebot, Emilio AL Gianicolo, Giuseppe Rainaldi, Maria Giovanna Trivella, Rosa Sicari

**Affiliations:** 1 CNR, Institute of Clinical Physiology, Pisa, Italy

## Abstract

Air pollution is increasingly recognized as an important and modifiable determinant of cardiovascular diseases in urban communities. The potential detrimental effects are both acute and chronic having a strong impact on morbidity and mortality. The acute exposure to pollutants has been linked to adverse cardiovascular events such as myocardial infarction, heart failure and life-threatening arrhythmias. The long-terms effects are related to the lifetime risk of death from cardiac causes. The WHO estimates that air pollution is responsible for 3 million premature deaths each year. The evidence supporting these data is very strong nonetheless, epidemiologic and observational data have the main limitation of imprecise measurements. Moreover, the lack of clinical experimental models makes it difficult to demonstrate the individual risk. The other limitation is related to the lack of a clear mechanism explaining the effects of pollution on cardiovascular mortality. In the present review we will explore the epidemiological, clinical and experimental evidence of the effects of ozone on cardiovascular diseases.

The pathophysiologic consequences of air pollutant exposures have been extensively investigated in pulmonary systems, and it is clear that some of the major components of air pollution (e.g. ozone and particulate matter) can initiate and exacerbate lung disease in humans [1]. It is possible that pulmonary oxidant stress mediated by particulate matter and/or ozone (O3) exposure can result in downstream perturbations in the cardiovasculature, as the pulmonary and cardiovascular systems are intricately associated, and it is well documented that specific environmental toxins (such as tobacco smoke [2]) introduced through the lungs can initiate and/or accelerate cardiovascular disease development. Indeed, several epidemiologic studies have proved that there is an association between PM and O3 and the increased incidence of cardiovascular morbidity and mortality [3]. Most of the evidence comes from studies of ambient particles concentrations. However, in Europe and elsewhere, the air pollution profile has gradually changed toward a more pronounced photochemical component. Ozone is one of the most toxic components of the photochemical air pollution mixture. Indeed, the biological basis for these observations has not been elucidated.

In the present review, the role of ozone as chemical molecule will be firstly considered. Secondly, pathogenetic mechanisms connecting the atmospheric ozone level and cardiovascular pathology will be examined. Thirdly, the literature relating hospitalization frequency, morbidity and mortality due to cardiovascular causes and ozone concentration will be studied. The correlation between ozone level and occurrence of acute myocardial infarction will be eventually discussed.

## Ozone

**Figure 1:**
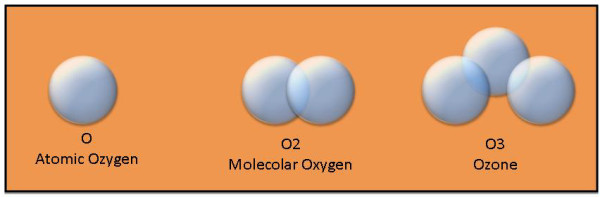
**Molecular structure of ozone**.

Ozone is a molecule consisting of three oxygen atoms, highly reactive and with a high oxidizing power. Ozone reacts with biomolecules to form ozonides and free radicals (figure 1). This reaction triggers an inflammatory response that conveys increased systemic oxidative stress, which has both pulmonary and cardiovascular effects. In nature, considerable concentrations of ozone are found at high altitude (15–60 Km high) within the so-called "ozonosphere", where it forms a layer protecting the earth from the sun's ultraviolet radiation.

On the other hand, in lower atmosphere layers, i.e. the so-called "troposphere" (less than 15 km high from the ground), ozone is naturally present at low concentrations, due to natural interchange with the stratosphere. This concentration, however, can rise in some area because of the so-called "photochemical smog", originating in those urban and suburban areas where other chemical pollutants (nitrogen oxides, hydrocarbons and VOCs, volatile organic compounds) combined with sun radiation might induce ozone formation, especially during the summer. Unlike "primary" pollutants, which are directly emitted by specific sources, ozone is a "secondary" pollutant, so it is difficult to determine the correlation with its precursors. The sources of ozone precursor pollutants are both anthropic (motor vehicles, combustion processes, thermoelectric plants, chemical solvents) and natural (woods and forests emit highly reactive VOCs, like terpens). To some extent, ozone is present in the troposphere because of the natural interrange with the stratosphere, and its concentration may vary between 20 and 80 μg/m3. Higher concentrations of ozone are caused by photochemical smog.

**Figure 2:**
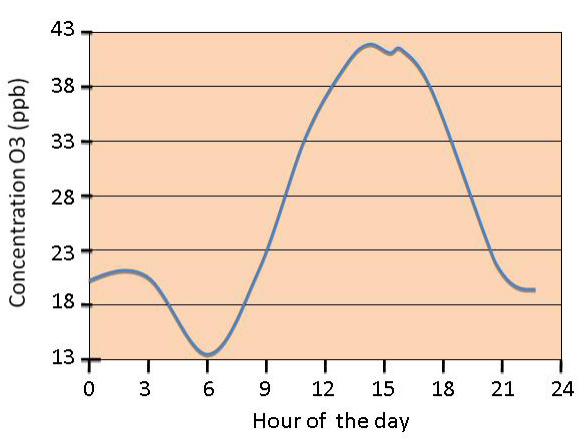
**O3 daily concentration redrawn from **[40].

Ozone concentrations are also influenced by different meteorological variables, such as sun radiation intensity and temperature. Therefore the presence of ozone varies during the day and different seasons. Ozone critical peak period is summer, when particular conditions like high pressure, low humidity, high temperature and scarce ventilation facilitate pollutant stagnation and accumulation. Moreover, high sun radiation triggers photochemical reactions, which are responsible for ozone formation. Generally, maximum values are reached during the hottest time of the day, from 12 to 6 pm, then decrease during night hours (figure 2). On the other hand, the lowest concentrations are recorded in winter, mainly because of the reduced sun radiation.

NOAEL (No Observable Adverse Effect Level) and LOAEL (Lowest Observed Adverse Effect Level) values have not been set for ozone. This means that apparently there is no threshold dose, below which no effects occur. To this end, it is very helpful to refer to the values provided by WHO, which sets an average value of 100–120 μg/m3 for the protection of global population. Moreover, this value is in line with the one set by Italian law.

If coordinates are to be defined for health protection, the following values can be referred to:

110 μg/m3 average on 8 hours (180 μg/m3 average on 1 hour) as health protection limit for subjects with a high sensitivity.

140 μg/m3 average on 8 hours (240 μg/m3 average on 1 hour) as health protection limit for subjects with a low sensitivity.

More than 220 μg/m3 average on 8 hours (more than 360 μg/m3 average on 1 hour) as health protection limit for the rest of the population.

Nonetheless, the remarkable influence of sporting activities on ozone potential effects must be taken into account: if outdoor sports are practiced more frequently, effects similar to the above mentioned ones can be observed, even at lower environmental concentrations of ozone. 110 μg/m3 average on 8 hours (180 μg/m3 average on 1 hour) as health protection limit.

## Biological effects of ozone on the cardiovascular system

**Figure 3:**
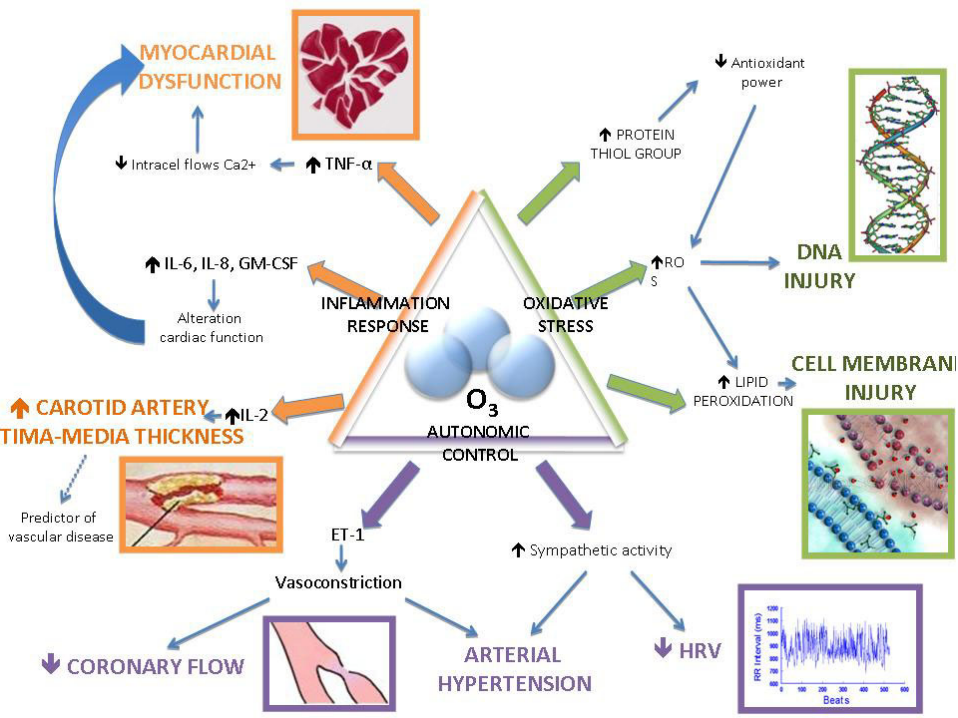
**Cellular effects of O3**.

Further studies aiming to demonstrate the correlation between the increased ozone concentration in air and cardiovascular diseases have been carried out. In particular, the effects on vascular tone, arterial pressure control, autonomic control of heart rate and serum concentration of inflammatory markers have been considered (Figure 3).

### Vasoconstriction

Evidence that ozone can influence macrovascular diameter and tone is found in a randomized, double-blind, crossover chamber study [4], which was recently reported. The study demonstrated that short term inhalation (2 hours) of both fine particulate air pollution (150 μg/m3) and ozone (120 ppb) at urban environment levels were associated with brachial artery narrowing. It is reasonable to suspect that the coronary vasculature may respond similarly to air pollution exposure because brachial and coronary reactivity are correlated. A reduction in coronary diameter of this relatively small magnitude (0.1 mm), however, would have minimal impact on healthy adults. Nonetheless, congruously with epidemiological findings that individuals at increased risk for acute air pollution-related cardiac events generally have pre-existing cardiovascular disease [5], this degree of sudden coronary vasoconstriction could promote cardiac ischemia in people with underlying flow-limiting obstructive lesions or could trigger instability of susceptible plaques [6]. Furthermore, the vasculature of patients with coronary risk factors is known to hyper-react to a variety of vasoconstrictors [7,8], which potentially increases their susceptibility for acute cardiac events after air pollution exposure. Additional investigations in coronary circulation and high-risk individuals are needed to confirm these hypotheses.

### Systemic arterial hypertension

Zanobetti et al. [9] have shown that air pollution may contribute to increased risk of cardiac morbidity and mortality in patients with pre-existing cardiac diseases, through increased peripheral blood pressure. In single pollutant models adjusting for all other non-pollutant factors but excluding PM2,5, higher resting diastolic blood pressure was significantly associated with 120 hours averages of ozone (2,7% increase; 95% CI 0,02 to 5,4).

Potential biological mechanisms for the vasoconstriction include a reflex increase in sympathetic nervous system activity via stimulation of pulmonary vagal afferents or an acute increase in vascular endothelin release, by direct mechanisms or via activation of oxidative stress pathways [4].

### Autonomic control of heart rate and arrhythmias

Reduced heart rate variability (HRV) is a predictor of increased risk for cardiovascular mortality and morbidity [10,11]. HRV may be a marker for poor health or an etiologic factor, representing a disturbance of the autonomic function, which increases the risk of cardiac events. Short-term increases in particle air pollution have been associated with a rise in daily cardiovascular mortality and morbidity in studies from cities throughout the industrialized world [12].

In their work, Gold et al. suggest that both particulate and O3 pollution may lead to short-term autonomic imbalance, reflected by changes in heart rate and HRV.

Holguin et al. have observed that increased ozone and fine particulate levels are especially related to a reduced high-frequency component of heart rate variability, this association being stronger in patients with arterial hypertension.

Whether or not pollution-related short-term changes in HRV or autonomic balance can partially account for associations between particulate or O3 [13] levels and cardiovascular morbidity or mortality is unknown. Between-person reduced HRV has been demonstrated to predict an increased risk for subsequent cardiac events in a population initially free of clinically apparent cardiac disease [11], increased mortality in the elderly [10], and sustained ventricular tachycardia [14]. In these between-person comparisons, altered HRV may contribute to mortality either as an etiologic factor or as a marker for poor health and subclinical coronary artery disease. If altered HRV is an etiologic factor for myocardial ischemia and fatal arrhythmias, it is hypothesized to work through sympathetic predominance and/or diminished parasympathetic tone.

An increase of air ozone levels has been also associated with a rise in risks related to the sopraventricular arrhythmia for the elderly [15].

Sarnat et al. observed odd ratio for having sopraventricular arrhythmia over the lenght of the protocol of 1,78% for 14,9 ppb increases in air pollution for moving average ozone concentration.

### Inflammatory markers

Recent studies support that O3 exposure mediates an inflammation response and increased oxidative stress in the cardiovascular system.

In vitro studies in isolated peripheral human blood mononuclear cells have shown that a significant relationship exists between O3 and increased lipid peroxidation and protein thiol group content [16]. Animal models also show that O3 exposure causes increased systemic oxidative stress [17].

Bocci et al. [18] have shown that ozone (O3) dissolved in the water of either plasma, serum or physiological saline generates reactive oxygen species (ROS), of which hydrogen peroxide (H2O2) can be unequivocally demonstrated. Lipids present in plasma, preferentially those present in lipoproteins, undergo peroxidation that is somewhat O3-dose dependent and can be observed by the measurement of thiobarbituric acid reactive substances. While the generation of H2O2 is crucial in activating both biochemical (hexose monophosphate shunt) and immunological (via the transcription factor NF-kB) mechanisms, the role of lipid oxidation products (LOP) remains to be investigated. In this study authors have also shown that there is a small but considerable induction of some cytokines (Tumor Necrosis Factor-alpha (TNF-a), Interferon-gamma (INF-g) [19], and interleukin-2 (IL-2)) when human blood is directly exposed to O3 concentrations up to 100 μg/ml per g of blood.

TNF-a is a cytokine involved in systemic inflammation and is a member of a group of cytokines that stimulate the acute phase reaction. Dysregulation and, in particular, overproduction of TNF have been implicated in heart failure. TNF-a can induce an immediate myocardial dysfunction, and it has been demonstrated that, in vitro, it can mitigate intra-cell flows of calcium ions [20].

IL-2 is a pro-inflammatory cytokine and its serum levels are associated with carotid artery intima-media thickness, a predictor of stroke and vascular disease.

Increased oxidant production can induce a spectrum of cytokines and associated mediators that can diffuse into the circulation and alter cardiac function. For example, in another study it has been demonstrated that human blood exposed to O3 leads to the release of other pro-inflammatory cytokine IL-6, IL-8, and granulocyte-monocyte colony-stimulating factor (GM-CSF) [21].

As far as pro-coagulant substances is concerned, Pekkenen et al. [22] have found no significant association between ozone levels and fibrinogen blood concentration.

## Epidemiologic studies

**Figure 4:**
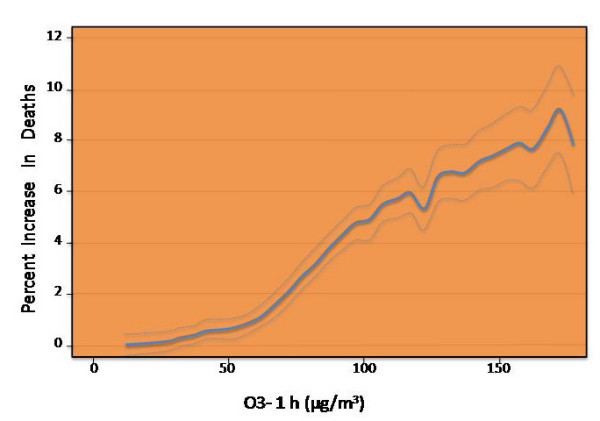
**O3 and mortality**. Dose-response of ozone 1 hour (average of lags 0 and 1) and daily number of deaths during the summer season) redrawn from [27].

In addition to its known pulmonary effects, O3 exposure in humans has been associated with increased hospital admissions related to cardiovascular complications, such as acute myocardial infarction, coronary atherosclerosis and pulmonary heart disease (figure 4). Early multicity time-series studies of O3 and mortality have estimated a broad range of effects; a 10 ppb increase in daily O3 was associated with increases in daily mortality of 2.84% (95% CI, 0.95–4.77%) for four European cities [23], 0.61% (95% CI, 0.38 to 1.60%) for seven Spanish cities [24], 1.40% (95% CI, 0.68–2.12%) for six French cities [25], and 0.43% (95% CI, 0.23–0.63%) for 80 US urban centers from 1987 to 1994. A recent multi-site time series study has provided strong evidence of an association between mortality and short-term exposure to O3. In a study that evaluated 95 communities within the U.S., there was a 0.52% (95% CI, 0.27–0.77%) increase in daily mortality for a 10-ppb increase in O3 concentration of the previous week. Moreover, these effects were still robust after the adjustment for PM and temperature [26].

Another large study, which assessed the effects of O3 exposure on daily total and cause-specific mortality from the 23 European cities, found that a 10 mg/m3 increase in the 1-h O3 concentration was associated with a 0.45% (95% CI, 0.17–0.52%) increase in the number of cardiovascular deaths, and the corresponding figures for the 8-h ozone were similar [27]. The associations with total mortality were independent of SO2 and particulate matter with areodynamic diameter less than 10 microm (PM10) but were somewhat confounded by NO2 and CO.

The association between daily variations in ozone and cause-specific mortality is been also investigated by Goldberg et al. in a study conducted in Montreal. For an increase in the 3-day running mean concentration of ozone of 21,3 μg/m3, the percentage of increase in daily deaths in the warm season is the following: non-accidental death, 3,3% (95% CI: 1.7, 5.0); cancer, 3.9% (95% CI:1.0, 6.91); cardiovascular diseases, 2.5% (95% CI:0.2, 5.0); and respiratory diseases, 6.6% (95% CI: 1.8, 11.8). These results were independent of the effects of other pollutants and were consistent with a log-linear response function. [28].

More recently, attention has been focused on the atmospheric pollution as possible specific trigger of acute myocardial infarction.

Ruidavets et al. [29] have conducted a study in which the association between hospitalizations for myocardial infarction in south-western France (Toulouse) from 1997 to 1999 and the atmospheric concentrations of nitrogen oxide, sulphure and ozone oxide has been remarked, using the case-crossover method. After correcting data according to temperature and humidity variables, the relative risk of acute infarction occurrence was significantly related to a rise in ozone concentration (median value of O3; 74.8 μg/m3) in the same day and the day after the episode (for an ozone increase of 5 μg/m3, the relative risk is 1,05 with p = 0,009 and 1,05 with p = 0,007, respectively). Instead, nitrogen and sulphur oxides have shown no statistically significant association. In Denver, which has a similar median level of O3 concentration (50.3 μg/m3), a 25th to 75th percentile change of ozone increased the risk of hospitalization for AMI and coronary atherosclerosis when the current day of O3 exposure and AMI admissions was considered [30]. In Barcelona (median value of O3 70.8 μg/m3), an increase of 100 μg/m3 of ozone air concentration was significantly associated (relative risk, 1.09) with increased cardiovascular mortality in the elderly during the summer [31]. In Mexico City (median O3 87.0 μg/m3), the mean concentration of O3 over a 2-day period was associated with a 1.8% increase in cardiovascular mortality [32]. In Hong Kong and London, with half the O3 concentration recorded in Toulouse (median O3 28.3 and 32.0 μg/m3, respectively), associations between O3 exposure and cardiac admissions were less consistent and remained uncertain [33]. Barnett et al. conducted a study in 7 cities within New Zealand and Australia, but no statistically significant correlation has been found between hospitalizations for acute myocardial infarction and ozone atmospheric levels.

Globally, the conflicting results could be due to the multiplicity of the studied health outcomes, the various types and levels of pollution, and investigation methodologies.

## Individual risks and experimental models: toward an environmental stress testing

As previously stated, one of the major problems related to the assessment of ozone impact on the individual subject is the need for a controlled experimental setting. The major limitation of these epidemiologic studies is the lack of adjustment for other major risk factors for CVD (including smoking, lack of physical activity, poor diet, and alcohol consumption). Another weakness of the reviewed mortality studies is the difficulty of accurately retrospectively assessing personal exposure. This remains a scientific challenge. Previous reports have demonstrated that the controlled exposure to ozone after a late airway response elicited by allergen challenge can potentiate the eosinophilic inflammatory response induced by the allergen challenge itself in subjects with mild atopic asthma [34]. This experimental setting may be theoretically transferred to assess the acute cardiovascular effects of ozone controlled exposure. Hypothetically, it is possible to design an environmental stress testing in which several imaging and functional biomarkers of early atherosclerotic damage are measured before and after a controlled ozone exposure. The environmental challenge would identify those patients at higher risk of developing overt coronary artery disease (Table 1).

**Table 1:** Experimental setting

	WHAT we have	WHAT we need
Design	Epidemiologic studies	Clinical Studies
Endpoints	Rate of disease occurrence	Death for CV causes
Sample size	Hundreds	Tens
Parameter	Population risk	Individual risk assessment

## Conclusion

The revision of literature underlines the association between ozone exposure and cardiovascular disease occurrence. A broader and deeper knowledge, yet incomplete, on the ozone biological effects and possible induced pathogenetic mechanisms supports the plausibility of this correlation. So far, mechanisms such as increase of oxidative stress, activation of a considerable systemic inflammatory response mediated by cytokines, modification of endothelial function and vascular vasomotricity, and alterations in autonomic control of cardiac frequency have been observed and associated to atmospheric ozone exposure.

In any case, it is commonly believed that biological effects of atmospheric pollution cannot be attributed to a single pollutant and it is not possible to classify some pollutants as more harmful than others. It is the atmospheric complex mix of different substances that, through common or different processes, determine the above mentioned cardiovascular effects. This statement helps understanding why no experimental study has been able to reproduce the environmental conditions, and why the results on the harmful effects of air pollution may extremely vary, thus influencing the correct scientific evaluation. For instance, the highest concentrations of ozone, which is formed by the effect of sun radiations on main pollutants (volatile hydrocarbons, nitro compounds, carbon monoxide), can be found at a considerable distance from pollution sources.

In a wide revision of literature, Maitre et al. [35] estimate that cardiovascular risk caused by atmospheric pollution is equivalent to the one related to moderate obesity (body mass index between 30 and 31,9 Kg/m2). Atmospheric pollution is now a ubiquitous problem regarding millions of people who are daily exposed. Therefore, even the slighter increase of relative risk corresponds to a great number of people who can show cardiovascular pathologic manifestations.

Another parameter influencing the sensitivity of the studies is the individual susceptibility to the effects of environmental pollutants, which remarkably varies among the examined population. Genetic predisposition, mediated by a different polymorphism of the genes involved in the oxidative and inflammatory response, can play a major role in influencing the effects of an interaction with atmospheric pollutants.

It has been recently reported that a reduction in exposure to ambient fine-particulate air pollution contributed to significant and measurable improvements in life expectancy in the United States [36]. Pope et al. noted that although decreases in fine particulate air pollution (PM2.5) could account for as much as 18% of the increase in life expectancy of approximately 2.74 years occurring in the United States between 1980 and 1999, other factors may be partly responsible [36]. The effects of ambient air pollution on population health can be addressed within the broader context of risk assessment and management. Population-health risk assessment involves the systematic evaluation of genetic, environmental and social determinants of health [37]. The evidence correlating pollution to cardiovascular disease is strong and ascertained by epidemiologic and clinical studies [38-40]. Primary and secondary prevention can be obtained only by reducing the atmospheric pollution rate, a social priority to be listed in the health policy agenda of the near future.

## Competing interests declaration

The authors declare that they have no competing interests.

## Authors' contributions

VS revised the literature and prepared the draft of the manuscript. EALG, GR, MGT revised the manuscript critically for important intellectual content. RS conceived of the review, and participated in its coordination and revised the final draft of the manuscript. All authors read and approved the final manuscript.

## References

[B1] Lippmann M, Frampton M, Schwartz J, Dockery D, Schlesinger R, Koutrakis P, Froines J, Nel A, Finkelstein J, Godleski J, Kaufman J, Koenig J, Larson T, Luchtel D, Liu LJ, Oberdorster G, Peters A, Sarnat J, Sioutas C, Suh H, Sullivan J, Utell M, Wichmann E, Zelikoff J (2003). The U.S. Environmental Protection Agency Particulate Matter Health Effects Research Centers Program: a midcourse report of status, progress, and plans. Environ Health Perspect.

[B2] Knight-Lozano, Knight-Lozano CA, Young CG, Burow DL, Hu ZY, Uyeminami D, Pinkerton KE, Ischiropoulos H, Ballinger SW (2002). Cigarette smoke exposure and hypercholesterolemia increase mitochondrial damage in cardiovascular tissues. Circulation.

[B3] Brook RD, Franklin B, Cascio W, Hong Y, Howard G, Lipsett M, Luepker R, Mittleman M, Samet J, Smith SC, Tager I, Expert Panel on Population and Prevention Science of the American Heart Association (2002). Air pollution and cardiovascular disease: a statement for healthcare professionals from the Expert Panel on Population and Prevention Science of the American Heart Association. Circulation.

[B4] Brook RD, Brook JR, Urch B, Vincent R, Rajagopalan S, Silverman F (2002). Inhalation of fine particulate air pollution and ozone causes acute arterial vasoconstriction in healthy adults. Circulation.

[B5] Krewski D, Burnett RT, Goldberg MS, Hoover BK, Siemiatycki J, Jerrett M, Abrahamowicz M, White WH (2000). Reanalysis of the Harvard Six Cities Study and the American Cancer Society Study on Particulate Air Pollution and Mortality.

[B6] Muller JE, Abela GS, Nesto RW, Tofler GH (1994). Triggers, acute risk factors and vulnerable plaques: the lexicon of a new frontier. J Am Coll Cardiol.

[B7] Lembo G, Vecchione C, Izzo R, Fratta L, Fontana D, Marino G, Pilato G, Trimarco B (2000). Noradrenergic vascular hyper-responsiveness in human hypertension is dependent on oxygen free radical impairment of nitric oxide activity. Circulation.

[B8] Cardillo C, Kilcoyne CM, Waclawiw M, Cannon RO, Panza JA (1999). Role of endothelin in the increased vascular tone of patients with essential hypertension. Hypertension.

[B9] Zanobetti A, Canner MJ, Stone PH, Schwartz J, Sher D, Eagan-Bengston E, Gates KA, Hartley LH, Suh H, Gold DR (2004). Ambient pollution and blood pressure in cardiac rehabilitation patients. Circulation.

[B10] Tsuji H, Venditti FJ, Manders ES, Evans JC, Larson MG, Feldman CL, Levy D (1994). Reduced heart rate variability and mortality risk in an elderly cohort: the Framingham Heart Study. Circulation.

[B11] Tsuji H, Larson MG, Venditti FJ, Manders ES, Evans JC, Feldman CL, Levy D (1996). Impact of reduced heart rate variability on risk for cardiac events: the Framingham Heart Study. Circulation.

[B12] Pope CA, Dockery DW, Schwartz J (1995). Review of epidemiologic evidenceof health effects of particulate air pollution. Inhal Toxicol.

[B13] Touloumi G, Katsouyanni K, Zmirou D, Schwartz J, Spix C, Deleon A, Tobias A, Quennel P, Rabczenko D, Bacharova L, Bisanti L, Vonk JM, Ponka A (1997). Short term effects of ambient oxidant exposure on mortality: a combined analysis within the APHEA Project. Am J Epidemiol.

[B14] Odemuyiwa O, Malik M, Farrell T, Bashir Y, Poloniecki J, Camm J (1991). Comparison of the predictive characteristics of heart rate variability index and left ventricular ejection fraction for all-cause mortality, arrhythmic events and sudden death after acute myocardial infarction. Am J Cardiol.

[B15] Sarnat SE, Suh HH, Coull BA, Schwartz J, Stone PH, Gold DR (2006). Ambient particulate air pollution and cardiac arrhythmia in a panel of older adults in Steubenville, Ohio. Occup Environ Med.

[B16] Larini A, Bocci V (2005). Effects of ozone on isolated peripheral blood mononuclear cells. Toxicol In Vitro.

[B17] Kodavanti UP, Schladweiler MC, Ledbetter AD, Watkinson WP, Campen MJ, Winsett DW, Richards JR, Crissman KM, Hatch GE, Costa DL (2000). The spontaneously hypertensive rat as a model of human cardiovascular disease: evidence of exacerbated cardiopulmonary injury and oxidative stress from inhaled emission particulate matter. Toxicol Appl Pharmacol.

[B18] Bocci Valacchi G, Corradeschi F, Aldinucci C, Silvestri S, Paccagnini E, Gerli R (1998). Studies on the biological effects of ozone: 7. Generation of reactive oxygen species (ROS) after exposure of human blood to ozone. J Biol Regul Homeost Agents.

[B19] Bocci V, Paulesu L (1990). Studies on the biological effects of ozone: 1. Induction of interferon gamma on human leucocytes. Haematologica.

[B20] Paulesu L, Luzzi E, Bocci V (1991). Studies on the biological effects of ozone: 2. Induction of tumor necrosis factor (TNF-alpha) on human leucocytes. Lymphokine Cytokine Res.

[B21] Bocci V, Luzzi E, Corradeschi F, Paulesu L, Rossi R, Cardaioli E, Di Simplicio P (1993). Studies on the biological effects of ozone: 4. Cytokine production and glutathione levels in human erythrocytes. J Biol Regul Homeost Agents.

[B22] Pekkanen J, Brunner EJ, Anderson HR, Tiittanen P, Atkinson RW (2000). Daily concentrations of air pollution and plasma fibrinogen in London. Occup Environ Med.

[B23] Touloumi G, Katsouyanni K, Zmirou D, Schwartz J, Spix C, de Leon AP, Tobias A, Quennel P, Rabczenko D, Bacharova L, Bisanti L, Vonk JM, Ponka A (1997). Short-term effects of ambient oxidant exposure on mortality: a combined analysis within the APHEA Project. Am J Epidemiol.

[B24] Saez M, Ballester F, Barceló MA, Pérez-Hoyos S, Bellido J, Tenías JM, Ocaña R, Figueiras A, Arribas F, Aragonés N, Tobías A, Cirera L, Cañada A, EMECAM (2002). A combined analysis of the short-term effects of photochemical air pollutants on mortality within the EMECAM project. Environ Health Perspect.

[B25] Le Tertre A, Quénel P, Eilstein D, Medina S, Prouvost H, Pascal L, Boumghar A, Saviuc P, Zeghnoun A, Filleul L, Declercq C, Cassadou S, Le Goaster C (2002). Short-term effects of air pollution on mortality in nine French cities. Arch Environ Health.

[B26] Bell ML, McDermott A, Zeger SL, Samet JM, Dominici F (2004). Ozone and short-term mortality in 95 US urban communities, 1987–2000. JAMA.

[B27] Gryparis A, Forsberg B, Katsouyanni K, Analitis A, Touloumi G, Schwartz J, Samoli E, Medina S, Anderson HR, Niciu EM, Wichmann HE, Kriz B, Kosnik M, Skorkovsky J, Vonk JM, Dörtbudak Z (2004). Acute effects of ozone on mortality from the "Air Pollution and Health: A European Approach" Project. Am J Respir Crit Care Med.

[B28] Goldberg MS, Burnett RT, Brook J, Bailar JC, Valois MF, Vincent R Associations between Daily Cause-specific Mortality and Concentrations of Ground-level Ozone in Montreal, Quebec. American Journal of Epidemiology.

[B29] Ruidavets JB, Cournot M, Cassadou S, Giroux M, Meybeck M, Ferrieres J (2005). Ozone air pollution is associated with acute myocardial infarction. Circulation.

[B30] Koken PJ, Piver WT, Ye F, Elixhauser A, Olsen LM, Portier CJ (2003). Temperature, air pollution, and hospitalization for cardiovascular diseases among elderly people in Denver. Environ Health Perspect.

[B31] Sunyer J, Castellsague J, Saez M, Tobias A, Anto JM (1996). Air pollution and mortality in Barcelona. J Epidemiol Community Health.

[B32] Borja-Aburto VH, Castillejos M, Gold DR, Bierzwinski S, Loomis D (1998). Mortality and ambient fine particles in south west Mexico City, 1993–1995. Environ Health Perspect.

[B33] Wong CM, Atkinson RW, Anderson HR, Hedley AJ, Ma S, Chau PY, Lam TH (2002). A tale of two cities: effects of air pollution on hospital admissions in Hong Kong and London compared. Environ Health Perspect.

[B34] Vagaggini B, Taccola M, Cianchetti S, Carnevali S, Bartoli ML, Bacci E, Dente FL, Di Franco A, Giannini D, Paggiaro PL (2002). Ozone exposure increases eosinophilic airway response induced by previous allergen challenge. Am J Respir Crit Care Med.

[B35] Maitre A, Bonneterre V, Huillard L, Sabatier P, de Gaudemaris R (2006). Impact of urban atmospheric pollution on coronary disease. Eur Heart J.

[B36] Pope CA, Ezzati M, Dockery DW (2009). Fine-particulate air pollution and life expectancy in the United States. N Engl J Med.

[B37] Krewski D (2009). Evaluating the effects of ambient air pollution on life expectancy. N Engl J Med.

[B38] Humblet O, Birnbaum L, Rimm E, Mittleman MA, Hauser R (2008). Dioxins and cardiovascular disease mortality. Environ Health Perspect.

[B39] Ljungman PL, Berglind N, Holmgren C, Gadler F, Edvardsson N, Pershagen G, Rosenqvist M, Sjögren B, Bellander T (2008). Rapid effects of air pollution on ventricular arrhythmias. Eur Heart J.

[B40] Gold RD, Litonjua A, Schwartz J, Lovett E, Larson A, Nearing B, Allen G, Verrier M, Cherry R, Verrier R (2000). Ambient Pollution and Heart Rate Variability. Circulation.

